# Indents

**Published:** 1915-10

**Authors:** 


### INDENTS

☞ **Brief Articles of Technical or Scientific Value will receive notice and comment. Contributions invited.**

*Endamoebae and Emetin.* Setting aside all issues as to the etiology, symptomatology, diagnosis, history, etc., and getting down to rock bottom on the endamoebae buccalis and the treatment of pyorrhea alveolaris with emetin hydrochlorid, the following facts may be of interest.

Together with Dr. F. E. Niece of New York, bacteriologist and head of the Claremont Laboratories, I have worked on 58 cases of pyorrhea, as follows: Males 34, average age forty-seven; females 24, average age forty-one —excluding two cases of eighteen and twenty years respectively. In all cases I used only Parke, Davis & Co.'s ampules of emetin hydrochlorid, 1 cc. 0.03 gm. (½ gr.). All treatments were given by hypodermic injection in the shoulder on three successive days. In each and every case, specimens were taken for microscopic examination before *every* injection. Endamoebae were always found when the case presented itself, and not found after the pus flow had stopped. The flow of pus stopped after using three injections in 50 cases, after four injections in six cases, and after six injections in one case.

In one case pus showed several days after the sixth injection; the patient became despondent and had the tooth, a sound canine, extracted elsewhere.

In no case did any local inflammation or reaction of any consequence appear. Injections were made in a girl of eighteen before going to school and in a lawyer before going to court. Inflammatory conditions in the mouth subsided remarkably after injections of emetin and the gums turned pink.

Owing to the microscopic work and the great number of slides examined, my opinion differs from that of Drs. Bass and Johns in that the endamoebae is not the *specific* cause of pyorrhea, especially as they acknowledge that pathogenic species of endamoebae may be found associated with harmless ones. It is my opinion that in future research work, the endamoebae will be found to be only a partially causative factor of pyorrhea, and that some other bacterial agent probably exerts just as great an influence. Still, the endamoebae may well be called a distinguishing diagnostic feature of pyorrhea. Emetin, absolutely aside from the endamoebae, proves greatly beneficial in reducing the inflammation, possibly by its known hemostatic action. It is my further opinion that practicing physicians lack the necessary implements for the local treatment of pyorrhea, and that for the practicing dentist the direct and intelligent use of the microscope, laboratory work, and research become imperative in the successful treatment of pyorrhea alveolaris.—R. C. Liemau, in *Cosmos*.

*Inlays by the Indirect Method.*

In making inlays by the indirect method I find that I can get much better margins by swaging into my copper amalgam cast a piece of 24K. 38 gauge gold plate and trim, leaving it slightly over-lapping, to the margins of the cavity in the cast. After trimming swage again, then build up with inlay wax to the desired contour; remove wax and gold matrix in situ and invest as usual. After the wax is burned out of the mould, cast the gold into the gold matrix almost boiling hot and it will fuse to the matrix. Now replace it in the amalgam cast, and swage it again and dress down to the margins and polish. After the inlay is seated into the cavity in the tooth you have a margin that can not be distinguished from a perfectly malleted gold foil filling. Another advantage in this method is that fact that you have pure soft gold in contact with the enamel of the tooth and can be easily burnished while harder alloyed gold can be used for the occlusal and contact portions of the inlay. I also find that the cervical margins of the inlay, made by the above method, are as beautiful as any other margin of the inlay. Dr. T. A. Leach, in *Digest*.

*Cause of Dental Caries.* Toward the end of an excellent report on the "Etiology of Dental Caries" presented to Section II of the Sixth International Dental Congress last August, Pickerill writes:

"To the etiology of dental caries a large number of factors may or do contribute. There are four chief factors, two attacking, carbohydrates and organisms; and two defensive, the resistance of the enamel surface and the protective action of the salivary secretion. Each of these may vary more or less, and so the possible permutation and combinations favoring caries are approximately twenty-four. But each of these factors is composed of a large number of contributing factors: The variations in the fermentability of the carbohydrates consumed, the number and type of organisms present, the different possible weaknesses of the enamel surface, and the varying amounts of the chemical constituents of saliva. In addition, there are the other systemic factors of internal secretions and hypernervous development. I have made a rough estimate of the possible permutations and combinations of all these factors, and find it is about 3,628,000. That is to say, in 3,600,000 cases of caries no two might have exactly the same combinations of causes * * *

There is no golden key to be discovered to unlock the riddle of dental caries."

With some of the above I am in agreement, but I feel convinced that until our knowledge of the laws of symbiosis, antibiosis, and commensalism, as practiced by the oral flora, is more exact, and our knowledge of the salivary secretion more complete, we can not be certain as to their influence in the causation of dental caries.

If it is assumed that the number of the micro-organisms in the oral cavity must be reduced, it is plain that this can be done by hygienic means, and by the exhibition of warm water or of weak acids which inhibit bacterial growth while doing no harm to Nasmyth's membrane or the enamel surfaces; acting on the hypothesis also that by using these measures we may destroy the beneficent influence they may possess, and perhaps alter or modify in some way the phagocytic action of cells inhabiting the oral cavity.

One thing would appear to be obvious—that the mechanical cleansing of the mouth and its contained organs is of the highest importance, that its sterilization is an impossibility, and probably an undesirability, and that the use of chemical agents and antiseptic remedies may, generally speaking, be not only valueless but sometimes actually harmful to the tissues, and should therefore be studiously avoided, unless their uses are distinctly indicated by signs and symptoms the significance of which are satisfactorily and convincingly established. — Professor Hopewell Smith, in *Dental Cosmos.*

*The Jacket Crown.* I believe this is an appliance the merits of which have not been sufficiently recognized, but it seems to me that it has a good many limitations. Dr. Capon says that any man of average ability with little effort and time should be able to make a jacket crown, but I think the operators who are successful in handling porcelain are men to whom this kind of crown most naturally appeals, and I know a great many who have conscientiously tried to use porcelain without satisfactory results. The technique of porcelain work, in my opinion, requires constant application and long experience before it can be perfected, which is one of its limitations. The field of application of this appliance, as shown by the essayist's pictures, is very wide. There has been some question in my mind in the last few years as to whether I have been doing the right and proper thing in placing jacket crowns on teeth with vital pulps. Dr. Capon, however, assures me that there will never be any trouble from covering vital teeth with jacket crowns. Some of my New York friends tell me that they have not for a number of years put any kind of crown over a vital tooth, for the reason that, when a vital tooth is covered and the natural influence of the fluids of the mouth is shut off from the tooth, it is placed in an abnormal condition and will undergo retrograde changes, and eventually, even though the patient may not know it, histological changes that will result in disease conditions. This I believe to be true if the tooth has been ground down very much. Teeth which have been liberally ground and then crowned are apt to suffer histological changes of the pulp, but Dr. Capon's method requires little radical grinding. It is quite likely, however, that in his practice conditions may be different from what they are in mine, because the technique which I have employed has always included the stripping of the enamel periphery of the tooth, leaving a well-defined shoulder at the free margin of the gum. In my early efforts I tried to go under the gum, but I found that this was unnecessary, or rather was detrimental to the gingival margin, as the gum tissue would not take kindly to the crown.—W. D. Tracy, in *Cosmos.*

*The Tooth Pulp.* There is only one absolutely safe pulp to leave in a tooth and that is the pulp in a tooth that is absolutely normal. A tooth begins to undergo pathological disturbances just as soon as it suffers from traumatism. It is not the quality of the cement, but the depredation in grinding and preparing the tooth for a crown that starts up a retrogressive metamorphosis in that tooth. Dr. Evans told us what would happen if we did this; that we will have a tooth with one-third or two-thirds of the pulp calcified and the rest doing service. Sometimes, but not always by any means. I could recite the history of more than one patient who had suffered many years from neuralgia because of the calcification of such pulps, with no infection and with no outward sign of trouble. This question of ''no trouble'' is one of observation; that is, you *think* a tooth has given no trouble. As has been well said, and as you will hear it said by Dr. Grieves that the best time to fill a root-canal is after a healthy pulp has been removed. The trouble in leaving pulps in teeth with the idea that one can drill through the crown later is this: Serious infection may occur long before patients know anything about it, and sometimes they never know anything about it, and when they do make the discovery there may be an apical disturbance which perhaps none of us can cure, which we know can not be cured by surgical operation—in other words, a permanent disturbance. I want to say in closing that we are not nearly so apt to see teeth that give trouble after our own operations as we are to see teeth that give trouble after our neighbors' operations.—Dr. Ottolengui, in *Cosmos.*

*Vital Pulps.* Pulp vitality is certainly a very vital question. Why is it that I take pride in doing this particular kind of work? I have practiced just twenty-five years and have been doing this work continuously, and I think I have saved myself a lot of trouble

The one reason why I have such regard for tne pulp when it is normal, is that I know nature has filled these canals better than I can do it. I showed you pictures of only a few cases of this work, and although I have treated hundreds of cases of various kinds, ranging from a single tooth to ten and twelve and fourteen in a mouth, the death of a pulp is rare. In seventy-five per cent. of these cases the crowns cover living pulps, and the percentage of trouble is so small that you may question my veracity if I gave the correct figures. You may extirpate all the pulps you want to, but I am proud of the fact that I do not do this unless I have to, and I am going to continue this practice, because I have proofs of the value of the work in these cases and in many others which I have not shown you.

The grinding of the teeth is frequently the cause of much trouble after the tooth has been crowned. Too many teeth are ground with a dry wheel, setting up an irritation which will eventually result in the death of the pulp. Keep plenty of water on the wheel, and do not push the grinding too hard, and if you can not prepare the tooth properly in this way, you can devitalize—you can always do that. The margins where the crown presses the gums are healthy in the majority of cases, because platinum is the medium; there is no getting away from the fact that this metal is most acceptable and non-irritating to the tissues.—Dr. Capon, in *Cosmos.*

*Use of Spence-Metal Models in Crown and Bridge Work.*

It has been my experience that Spence-metal is a most valuable material in producing models to be used in the construction of bridge work where a very accurate bite is desired. Models made of this substance are much sharper than plaster and when mounted upon the articulator, stand hard usage without bruising or disturbing the occlusal surfaces of the model teeth, so apt to occur when plaster models are used.

The method of procedure is as follows: Place the abutment pieces (crowns or inlays) in position, warm a sheet of Detroit modeling compound and secure a bite by having the patient close the teeth firmly upon it. After this is accomplished, take a plaster impression of the abutment pieces and adjoining teeth, and a plaster impression of the teeth to be articulated. As soon as this is finished, the abutment pieces are placed in the impression and a thin coating of moldine clay mixed with water is painted over the cavo-surfaces of the inlays, or in the case of shell crowns, they may be partially filled with moldine, thus avoiding undercuts, which would prevent their removal from the model. The impressions are then well saturated with kerosene, and strips of moldine clay built up around their margins to confine the Spence-metal and prevent it from overflowing. We are now ready to pour the Spence-metal, which has previously been melted in a small granite iron sauce pan over a slow flame.

Impressions must be poured as soon after they are taken as possible. Should they be allowed to stand long enough to dry out, it will be necessary to thoroughly saturate them with kerosene to prevent the Spence-metal from adhering to the plaster. Care must be exercised in melting not to overheat, since the sulphur in the compound will be driven off, producing an objectionable odor and making the metal stiff like batter. If overheated, remove from the flame and wait until the molten mass thins, and the bubbles have nearly disappeared. When the consistency of the material is smooth and fluid, resembling ink, pour into the impressions. Hardening will take place immediately. Now place them in water, separate models from the impressions, scrub them and articulate. The abutment pieces should be loosened so that their removal may be easily accomplished, thus making it possible to remove the waxed bridge work for investing.

Success with this method is assured, because after the piece is soldered, it may be again placed upon the model and any slight adjustment of the articulation made.—Dr. L. G. Watkins, in *Summary*.

*Dangers of Local Anesthesia.* These are chiefly due to toxic doses of the drug given especially in concentrated solution which are rapidly thrown into the blood stream; idiosyncrasy plays a part, but a comparatively small one.

One must here consider the dangers consequent to errors in technique, for if an infection occur it is not the fault of the anesthetic but the fault of the anesthetist.

One must consider the age of the individual, remembering that children and old people are more susceptible. Before using any toxic drug one should become familiar with its physiological action and toxicology, including the physiological and chemical antidote.

As novocain is almost identical with cocain we will consider the symptoms of systemic poisoning from cocain. Housmann, who has made an exhaustive study of the drug, says that the lightest form is manifested by sudden dizziness, usually very shortly after its injection, which rapidly subsides. This attack may continue, and pass into a state of collapse, with small, rapid pulse, cold extremities, irregular and labored breathing and cold sweat. And in more severe cases loss of consciousness, which in a few hours advances to a very weakened condition; vomiting is not infrequently observed. It is probable that the condition of cerebral anemia induced by the drug and psychic shock is responsible for some of these transient symptoms. In a higher grade of toxemia the symptoms represent a cerebral cortex irritation, the patients are unduly excited, laugh, seem unnaturally happy, talk rapidly upon various topics, may be delirious or have hallucinations. All kinds of subjective symptoms are present, dryness of the throat, loss of sight, taste, and hearing; the pupils are wide and inactive.

In very severe cases, epileptiform convulsions occur, with exophthalmos and loss of consciousness, with loss of all reflexes, coma, and death by paralysis of the respiratory center. As before stated, it is the presence in a given volume of circulating blood of a concentrated solution which is carried to the vital centers, which produces the dangerous symptoms, and the avoidance of that is to be sought by the following means, except in cases of idiosyncrasy, and that we can not control except by the history of a previous poisoning:

*First:* By the use of weak solutions of novocain. A solution stronger than two per cent. should not be used, and usually one-half per cent, is strong enough except in the most difficult cases.

*Second:* The injection should not be made into a vein. This can be avoided by moving the needle backward and forward during the injection.

*Third:* The addition of suprarenal extract to the solution. Klapp, Braun, and others have shown that suprarenal extract greatly retards its absorption, enhances the anesthetic by prolonging its action, and as it intensifies the anesthesia much weaker solutions can be used; furthermore it produces a more or less bloodless field.

Too much suprarenal extract, however, should not be used, as symptoms such as feelings of oppression, palpitation, and increase in pulse rate, rapid and deep breathing may be caused by it. These, however, are evanescent and not serious, as its injection beneath the skin does not raise blood pressure as it does when it is injected into a vein, and follows the injection of ½cc. or more.

*Fourth:* One should not inject the anesthetic solution into an abscess for by so doing the septic material and organisms may be forced from their comparatively benign location into the blood or lymph-stream and set up a similar pathological condition in other parts of the body. Syncope not infrequently ensues at the beginning or even before the operation. This psychic shock presents symptoms which are very similar to a light toxemia from the drug, and can be avoided by having the patient in an horizontal position in the chair or on the table, and the avoidance of fear by morphine-scopolamine, if necessary, for Crile has shown that this psychic shock is deleterious. One can usually distinguish between them, as fainting is usually preceded by some nausea, a blanched face or cold perspiration. Nervous, fearful patients, if they will not yield to reason and more or less hypnotic influence, may be given chloral hydrate with potassium bromide each xv gr. an hour or so before the operation. Morphine gr. 1-6 with scopolamine gr. 1-150 may also be given if the case warrants it one hour before. This is frequently done preceding the more serious operations, such as resections of the jaws, removal of carcinomatous growths of the mouth, etc.

If the prodomal symptoms of syncope develop, all that is necessary is to place the patient in a horizontal position, or even place the head lower than the plane of the body, and ammonia, smelling-salts, or amyl-nitrate may be administered by inhallation if necessary.

If it is evident by a more rapid onset and increasing depth of toxemia, even with the patient in the horizontal position, that the trouble is due to the poisonous effects of the anesthetic the treatment should be symptomatic, as there is no chemical antidote for novocain.

The cerebral anemia is counteracted by lowering the head and breaking a pearl of amyl-nitrite in a piece of gauze, allowing the patient to inhale it.

If convulsions supervene ether narcosis is indicated, to be stopped just as soon as the convulsions are over; if longer continued, it is harmful.

The collapse which follows the convulsions indicates the need of stimulants; ½cc. of camphorated oil may be injected subcutaneously, or as Fischer advises, 5 to 7 drops of Validol internally, black coffee also per rectum or per os, if the patient can swallow, and rubbing the skin and keeping the body warm should not be neglected.—Dr. H. A. Potts, in *Review*.

*Accidents in Operating.* We have all heard of jaws being broken or dislocated during the extraction of teeth. I know of no authentic case of a fracture of the jaw during extraction, but every dentist knows of cases of fracture of the alveolar process. The most serious of these cases seems to be in the region of the upper second and third molars. The whole tuberosity loosens up with the teeth. In the majority of cases it is wise to immediately dissect the fragment out and bring the soft parts together with sutures. Occasionally the ridge holding the six lower anterior teeth loosens in attempts to remove a tooth. Dislocations do occur during extraction and sometimes during heavy pressure in operating. The one consoling fact is that the jaw which readily dislocates has done it before and can easily be restored. Wounds to soft tissues by slipping forceps, elevators, excavators and engine instruments heal rapidly. The most dangerous instrument is a sharp-edged stone or saw in the handpiece. I once saw a student undertake to demonstrate to another how to cut a bicuspid tooth off with a 3/4-inch circular saw. In a second or two the saw had cut through the cheek and back into the mouth again in a new place, just sparing the facial artery. The wounds fortunately healed by first intention, leaving little or no scar. While such accidents seem serious at the time they are not to be compared in seriousness from a dento-legal aspect with extracting the wrong tooth, allowing a tooth, a crown, or a partial denture to be swallowed. Almost everybody who has lost many teeth has had the wrong one extracted sometime or knows of some one who has had the wrong one extracted. A dentist who has not done such a thing can hardly conceive of how it could happen. Patients often make their own diagnosis as to the tooth causing pain and order the dentist to extract it; some dentists are foolish enough to do so, only later to be told that they extracted the wrong tooth. Many suits for damages have occurred because of bad diagnosis and misunderstandings between dentists and patients. No one is insured against making a mistake; the wrong eye has been removed, the wrong kidney operated upon, the wrong antrum opened and the wrong wisdom tooth removed even in the presence of a radiograph. Only the inexperienced, foolish and ignorant are sure of things. Very good advice was given by Dr. Ottofy, who said: "Never begin an operation without first going over in the mind what accidents might occur." If anything untoward should occur the operator would not be taken by surprise and would be better prepared for an emergency. "No truly great man is ever surprised."—A. E. Webster, *Dominion Dental Journal.*

*Infected Third Molar Sockets.* The following treatment is suggested: First of all the use of sterilized tepid water, or, if preferred, with the addition of normal salt or boric acid. After washing it thoroughly the socket may require curettement to insure the removal of any abnormal growth resulting from infection and inability of the tissue to heal normally. In cases of continued pain, camphorated oil gives relief in mild cases, but if extreme anodynes are necessary, one-eighth grain of morphine incorporated with balsam of Peru and iodoform (odorless) made into a paste, may be carried to the floor of the socket in a pledget of cotton, and sealed with Fletcher's carbolized resin and cotton, subsequently reducing the size of the dressing as the socket closes. In case of enlarged sub-maxillary and parotid glands the use of one part of tincture of opium and two parts lead water applied on flannel and wound with a hot cloth for an efficient (external) application for the relief of pain.—Dr. Hopkins, *Pacific Gazette.*

*To Remove Tin from Plates.* Small particles of tin adhering to vulcanite plates can be easily removed by mixing mercury with enough alloy to keep it from flowing and rubbing it over the plate under fingers.—Dr. Hopkins, in *Pacific Gazette.*

*A Full Practice.* I recall a remark made in this connection by Dr. Atkinson, who in his very forceful way said, ''A full practice is a —— bad practice.'' In such a practice it is nearly impossible for the average dentist to give the necessary time to his patients. Yet, few patients do not enable one to pay his running expenses, and therefore he has to have as many as he can get. There is another bad feature that has become evident in late years—that is, that every dentist is trying to command very high fees, especially the younger men. Wealthy people can pay them, but how can the poorer people pay the prices that are demanded? There is hardly a young man graduating from a college today who is not looking forward to the time when he will have a well-paying practice, and one of the brags that we hear frequently from a dentist is that he has received fifteen or twenty dollars for a crown. Poor people can not pay such fees, and the reason why so many of these operations turn out badly is because the dentist is simply working to get the work finished and receive his money.—Dr. James McManus, in *Cosmos*.

*Cause of Pyorrhea Pockets.* In a large percentage of cases, I believe that some initial lesion is the beginning of the pus pocket. When there is an irritant that will produce inflammation, germs, which are always present, will carry on the work, and when inflammation has developed sufficiently it will go on independently of the original cause. It takes very little to produce a pus pocket sometimes. I recall a case where a tiny piece of rubber dam had been left under the margin of the gum on a central incisor. In packing gold into a cavity the dentist had caught the dam between the gold and the margin of the cavity, and a year and a half later a pus pocket with considerable loss of the membrane had resulted. The peridental membrane is a tissue that when once destroyed does not reproduce itself.—Dr. N. A. Stanley, in *Cosmos*.

*Adjustment of Clasps.* Dr. Weinstein says the way to compensate the pressure of mastication is to make the vulcanite saddles, cut out the under side, put in modeling composition, have the patient bite upon it, and then substitute vulcanite for the modeling composition;—that, in masticating on a denture thus made, the denture will spring to place as soon as the pressure is removed. When clasps are used in connection with that method the clasps will also spring up on the teeth, and this constant up-and-down action will surely wear the teeth. With the Roach attachment none of these occurrences would matter.—Dr. Rule, in *Cosmos*.

*Right to Recover for Dental Services.* There can be no recovery in New York for dental services where plaintiff fails to show that when he performed the services he was duly licensed to practice his profession, according to a recent decision of the New York Supreme Court handed down in the case of O'Beirne vs. Carey, 150 New York Supplement 666. This decision means more than that an unlicensed dentist can not recover for work done; it means that even a licensed dentist must affirmatively establish the fact that he has been duly registered. In the latter case, the dentist's assumption that the patient who is sued will not controvert the fact that a license has been issued is apt to result in an adverse judgment, unless respect is paid for the decision announced in the cited case.—*Dental Digest.*

